# Some thoughts on integrity management of mounded storage tanks

**DOI:** 10.1016/j.heliyon.2023.e22254

**Published:** 2023-11-14

**Authors:** Xiaowei Li, Chenyang Du, Chang Liu, Ce Song, Jun Yuan, Jianyu Lu, Yanchao Xin

**Affiliations:** China Special Equipment Inspection and Research Institute, Beijing, 100029, China

**Keywords:** Mounded storage tank, Risk factor, Integrity management, Integrity assessment, Risk assessment

## Abstract

In the domestic petrochemical industry, mounded storage tanks (MSTs) are widely used to store hazardous chemicals. The shell of the mounded storage tank is completely covered by soil to effectively mitigate the effect of the external environment and prevent thermal-expansion-induced explosion of the stored material. Because mounded storage tanks are mostly underground, they are highly safe, provide effective land utilization, and are highly energy efficient. Furthermore, the impact radius in case of an explosion is less than that of aboveground tanks. However, adequate regulations and standards for safety management are yet to be established. This study established a novel method for the integrity management of mounded storage tanks through database construction, risk assessment, applicability monitoring, and testing. At the same time, the risk assessment method for mounded storage tank characteristics is constructed for the first time.

## 1 Introduction

Risks, regardless of human errors or inherent factors [[1], [2], [3], [4]], can damage the integrity of equipment and the system. Integrity management can reduce risks and is a type of managerial activity that is performed to continuously identify and evaluate equipment-related risk factors. Various measures are subsequently initiated to ensure the safe and economic operation of equipment.

Integrity management is a novel management style that originated in the United States. Integrity management was first used to research the frequent failure of long-distance oil and gas pipelines. With the popularization of integrity, integrity management has gradually been adopted in various applications [[5], [6], [7], [8]]. Integrity management closely follows the development of science and technology and is consistent with the emerging technical concepts such as blockchain [9,10] and Industry 4.0 [11,12]. Thus, integrity management has universal applicability and strong vitality and has developed rapidly. For example, researchers at the Centre for Risk, Integrity, and Safety Engineering (C-RISE) of Memorial University in Canada have conducted in-depth research on the safety risk consequences and dynamic risk assessment methods of pipelines [[13], [14], [15], [16], [17], [18]]. Their achievements have played a crucial role in promoting pipeline integrity management and risk assessment and their adoption by the industry. Rachman et al. [19] used machine learning in pipeline integrity management. Sebastian Thöns [20] investigated the framework of quantifying the value of structural health monitoring (SHM) under structural risk and system integrity management and evaluated the importance of SHM in integrity management. Afangide et al. [21] proposed a quantitative method for health monitoring of submarine pipelines to effectively improve the existing pipeline integrity management model and reduce the cost of integrity management. The results of the studies reveal that the development of integrity management in special equipment is closely associated with the latest technology development trend. The latest technology should be used to reduce the operation risk of special equipment and the cost of safety management.

Integrity management was introduced in China in 2000. Initially, integrity management was confined to long-distance oil and gas pipelines, which subsequently led to the integrity management of gas pipelines, vessels, storage tanks, and even complete sets of petrochemical equipment [[22], [23], [24], [25], [26]]. China has established standards including GB 32167–2015 *Oil and gas pipeline integrity management specification* [27] and GB/T 37327–2019 *Integrity management of atmospheric storage banks* [28].

In the petrochemical industry, MSTs are typically used to store raw materials and finished or semi-finished goods. Because such materials are highly flammable and explosive, the location of an MST is a key risk point. Moreover, considering MST installation is a critical procedure, their conventional management is not applicable. Therefore, establishing an integrity management system for full-cycle management of an MST is critical.

This study is the first to develop the integrity management system framework of the MSTs, explain their integrity evaluation method, and propose the risk evaluation method according to their characteristics. Finally, the study discusses its shortcomings and offers future recommendations.

## 2 Characteristics of MST integrity

**Fig. 1:**
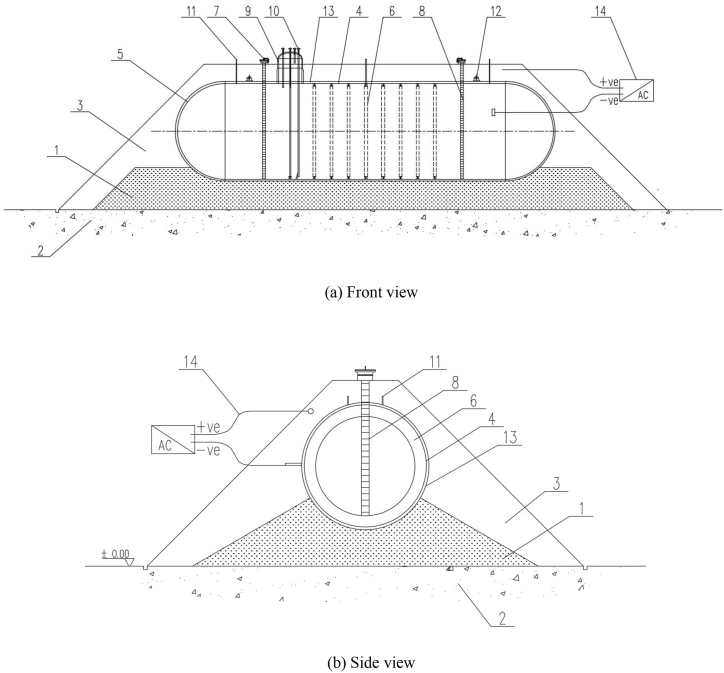
Structural schematic diagram of an MST. Notes: 1—Sand bed; 2—Flat ground; 3—Overburden soil layer; 4—Cylinder; 5—Seal head; 6—Reinforcing ring in tank; 7—Man-hole; 8—Ladder in tank; 9—Air chamber; 10—Process pipe joint; 11—Settlement monitoring board; 12—Lifting lug; 13—Anticorrosive coating; 14—Cathodic protection system.

An MST, also known as a mounded vessel or bullet tank, is a common type of MST with a long cylindrical shape. Generally, the tank body is placed on a sand bed, with its outer surface completely covered by soil and only the relevant nozzles (for feeding and discharging, unloading, sewage disposal), manholes, gauge pipe orifices, and safety accessories outside the overburden soil layer. Figures [1(a, b)] display the schematic of the MST.

MSTs are generally fabricated of steel for pressure vessels, such as carbon steel, low alloy steel or austenitic stainless steel with the working pressure between −0.1 and 10 MPa. The working temperature is between −20 and 60 °C. The internal medium is a mildly hazardous or nontoxic medium specified in HG/T20660 [29].

Generally, an MST is placed directly on the sand bed, beneath which is the flat ground with uniform settlement. The tank is covered by soil and is equipped with the process and control instrumentation nozzles, inspection and overhaul structures, and safety, firefighting, and supporting components. Considering the special method required for the installation of the tank, conducting routine inspection and detection is difficult. An MST is typically equipped with devices for monitoring wall thickness, leakage, cathode protection, settlement, temperature, pressure, liquid level, and overburden soil environment to evaluate the operating safety of the tank.

Table 1 summarizes the risk factors related to the integrity management MST based on the structure, sand bed, overburden soil layer, instrumentation, process, and control system.

**Table 1:** Key risk factors for the integrity management of mounded storage tanks.

Main Components	Risk Factors	Factor Range
Tank body	Brittle rupture Ductile fracture Plastic instability Corrosion thinning Stress corrosion cracking Vibration fatigue	Fracture toughness, structural stress concentration, and residual stress of the material Design calculation (including tank strength, stiffness, and stability under external load, etc.) Overload, component stress change Anticorrosive coating of the tank body, corrosion allowance for the material, and overburden impurities Medium-material compatibility Rotating equipment connected to nozzles
External anticorrosive coating	Anticorrosive coating failure	Material of anticorrosive coating, brushing quality, cathodic protection, etc.
Sand bed foundation	Non-uniform settlement	Thickness of sand bed, bearing capacity of pile foundation, and uniform settlement
Overburden soil layer	Overburden soil layer instability Waterlogging in overburden soil layer Corrosiveness of overburden soil layer (soil, microbial corrosion)	Slope of overburden soil layer, surface vegetation Open channel, tank, pool, and draining pump set up at the bottom of the sand bed Selection, cleaning, and desalination of overburden soil
Accessory equipment	Corrosion of pipelines, valves, etc. Flange seal failure Weld failure in supporting seat and reinforcing ring	Anticorrosion quality of pipelines and valves Pressure class, gasket, fastener, and leakproof structure of the flange Weld failure in the supporting seat and reinforcing ring
Instrument and monitoring system	Instrument failure Aging and failure of monitoring facilities	instruments Aging and failure of leakage, settlement and cathodic protection monitoring facilities
Firefighting system	Fire sprinkler system failure	Failure of fire sprinkler facilities
Personnel operation	Misoperation	Misoperation

## 3 Integrity management system of MSTs

MST integrity management denotes technical improvement measures adopted with standard vessel management so that the MST remains intact throughout its life cycle. First, the integrity of the MST should remain intact in its life cycle in terms of its design, construction, operation, maintenance and even scrapping. Second, establishing a corresponding technical system and management system is necessary to ensure that the MST is always under control and stays intact throughout its life cycle (see Fig. 1).

**Fig. 2:**
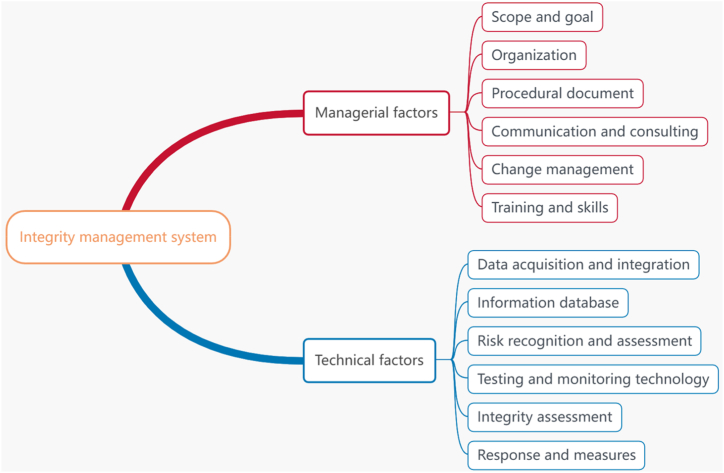
Integrity management system of a mounded storage tank.

According to MST definition and the integrity management of atmospheric storage tanks, oil–gas pipelines and liquefied natural gas tanks, the integrity management system of MST depends on managerial and technical factors. Managerial factors are categorized into a document system and a management platform including scope and goal, organization, management framework and procedural documents, operating procedures, personnel training, and change management. Technical factors include data collection and integration, information database, risk assessment technology, detection technology and integrity evaluation, monitoring technology, risk-based detection technology, usability evaluation technology, emergency management, failure management, deactivation, and scrapping. Neither managerial factors nor technical factors are constant and require adjustment according to the situation, technical development, and system effectiveness. The system can be either simple or complex. The objective is to optimize the integrity management of MSTs and cost efficiency of management. Fig. 2 displays the framework of the integrity management system for an MST.

The scope and goal are designed to determine the coverage of integrity management, the acceptable level of risks, the control level of failure events, and other objectives of integrity management. An organization is set up to ensure the smooth implementation of integrity management, with job responsibilities clearly defined for personnel. Procedural documents refer to related files and records used to ensure the effective implementation of integrity management and process control. Communication and consulting are necessary for ensuring the smooth implementation of integrity management, with a communication mechanism established for the exchange of ideas with the staff, management, clients, relevant departments, and emergency response team. Change management is conducted to ensure that the tank can effectively identify the effect of any change on its operation before the change is implemented. The change is subsequently recorded and evaluated. Training and skills are primarily focused on improving the skill level of personnel in various integrity management positions according to a training program.

Among technical factors, data collection and integration refer to collecting, analyzing, and collating the life-cycle data of equipment, and subsequently establishing a corresponding information database for equipment maintenance. Risk identification and assessment consists of identifying equipment risks based on use management, medium, operating conditions, and damage mode, followed by the determination of the risk level through corresponding risk assessment. Detection and monitoring technology is used to check and reduce risks. Integrity evaluation is designed to evaluate any identified risk factor to examine equipment integrity. Response and measures are response procedures and emergency measures formulated to cope with any emergency.

The design of integrity management is dynamically and cyclically implemented throughout the life cycle of an MST. An integrity management information system can be established with the evaluation of the effectiveness on a regular basis to ensure continuous improvement of the integrity management method in the implementation process, identification and solution of problems, such as equipment aging, short-term failure of safety measures, operation change, maintenance failure, misoperation, and low skill level, as well as for periodic inspection of the structural integrity, functional integrity, and operational integrity of the MST.

## 4 Methods for the integrity assessment of MSTs

The integrity assessment of MSTs is based on risk management. The integrity assessment technology is widely used to evaluate the risks of the tank body and ancillary facilities. Furthermore, the assessment methods proposed for its supporting systems (e.g., machinery, instrument control, monitoring, fire protection, etc.) are used to comprehensively evaluate the integrity of the MST systems. Next, a proper detection and monitoring method and operation strategy can be selected to determine the key points of daily maintenance and monitoring according to the risk level to realize the safe and reliable long-term operation of the MST system.

The integrity assessment of MSTs should be supported by a complete set of technical standards and basic databases to integrate risk assessment, monitoring technology, detection technology, integrity/applicability assessment, and emergency response into daily management and regular evaluation [30]. To realize this integration process, an appropriate monitoring and detection evaluation technique can be used for the risk and applicability assessment of the MST systems.

### 4.1 Information database of the MST system

The information database of the MST systems comprises essential data and information that reflect the basic information, safety status, and risk sources of the MST system, which are as follows:

a) The design, manufacturing, installation, operation, maintenance, monitoring, detection, and other related information of the MST, as well as information related to abnormal conditions;
b) Function degradation and failure information, including corrosion, cracking, brittle fracture, fatigue, and mechanical damage, as well as inducing factors and consequences;
c) Information about its harmful effect on the personnel, environment, and operations.

The information in the information database originates from the design drawings, construction records, material quality certificates, manufacturing quality certificates, technical operation parameters, acceptance reports, equipment operation and management plans, emergency treatment schemes, technical evaluation reports, operation specifications and relevant standards, as well as expertise. During implementation, the list of integrity assessment techniques should be dynamically updated to ensure that the historical data and changing trends of specific indicators are accurately reflected. The information database should cover all information essential for the implementation of integrity management and decision-making for MSTs as it is the supporting platform for the implementation of integrity assessment.

### 4.2 Methods for the risk assessment of MSTs

Based on its design parameters, an MST is a pressure vessel. Currently, China has established guidelines for the risk-based implementation of inspections on pressure equipment [31], including qualitative and quantitative risk assessments. However, because of the special installation form of mounded vessels, inaccurate results are obtained if a standard risk assessment method is adopted.

First, the MST is fully covered by silty soil, and its damage modes differ from those of conventional pressure vessels. Corrosion thinning, corrosion cracking, mechanical damage, and other damage (e.g., low-temperature brittle fracture) are the common modes of damage to the MST. The outer part of the tank is susceptible to atmospheric, soil, and microbial corrosion. The level of corrosion depends on the composition and quality of silty soil. Second, because the tank surface is covered by silty soil, its failure or explosion has less severe consequences than that in the air because soil absorbs some explosion energy attenuating the explosion shock wave to a certain degree. Studies [[32], [33], [34], [35], [36], [37]] have revealed that soil limits the impact range of equipment explosion, that is, equipment explosion in soil is merely 40.7 % and 12.9 % of aerial explosion in terms of safety radius for humans and safe distance for buildings, respectively. This phenomenon reveals that the impact radius increases and risk assessment becomes inaccurate if the safety consequences of the MST are estimated according to the GB/T 26610 standard [31].

For an MST, a special risk assessment method should be developed in accordance with its characteristics. In this study, risk classification indexes and relevant calculation methods were established according to the inherent characteristics, serviceability, and risk factors for the MST, as presented in Table 2. Additionally, a quantitative evaluation method was developed for each index (Table 3). Failure possibility and failure consequence were graded using the quantitative evaluation methods of risk classification presented in Table 2, Table 3 The scoring results were used for interval division, and a 3 × 3 risk matrix was established to determine three risk levels, namely high, medium, and low. Finally, the risk level of the MST was dynamically adjusted according to the relevant basic, testing, and monitoring information available in the information database.

**Table 2:** Risk classification indexes and calculation method for MST.

Total Score	Index Calculation for Each Factor				Sub-item Index	
	Index Name	Symbol	Weight	Calculation Method	Sub-item Index	Symbol
Score of failure possibility FP = M + E + S + I	Material	M	W_M_	M=(M1+M2) × W_M_	Material suitability	M1
					Internal and external anticorrosive coatings	M2
	Service environment	E	W_E_	E=(E1+E2+E3+E4+E5+E6+E7) × W_E_	Soil corrosiveness	E1
					Corrosiveness of internal medium	E2
					Cracking behavior of internal medium	E3
					Foundation settlement	E4
					Soil waterlogging/instability	E5
					Low-temperature brittle fracture	E6
					Ambient vibration source	E7
	Operating state	S	W_S_	S=(S1+S2) × W_S_	Service length	S1
					Historical failure	S2
	Testing, monitoring	I	W_I_	I=(I1+I2+I3) × W_I_	Overall inspection of inner and outer sides	I1
					Risk-based inspection	I2
					Monitoring results	I3
Score of failure consequence FC=B + V + P + L	Flammability and explosiveness of medium	B	W_B_	B=B1 × W_B_	Flammability and explosiveness of medium	B1
	Tank volume	V	W_V_	V=V1 × W_V_	Tank volume	V1
	Economic impact	P	W_P_	P=P1 × W_P_	Economic impact	P1
	Environmental effect	L	W_L_	L = L1 × W_L_	Economic impact	L1

**Table 3:** Quantitative evaluation method for each risk classification index of the mounded storage tank.

Sub-item Index	Symbol	Highest Score	Quantitative evaluation method for indexes (the stronger the impact, the higher the score)
Material suitability	M1	20	Material suitability is mainly determined in accordance with the operating conditions and environment of materials and equipment, as well as the relevant information of internal medium. The higher the score, the poorer is the suitability, and the higher is the risk of failure. Suitability is determined by GB/T 30579 [39], engineering practice experience and published research results. The score ranges from 0 to 20.
Internal and external anticorrosive coatings	M2	10	It is determined by the availability of internal and external anticorrosive coatings, the anticorrosive effect of internal and external anticorrosive coatings on the medium and soil, and the current state of the anticorrosive layer. Currently, UV-curing technology [40] has been applied as an external anticorrosive coating to some mounded equipment, i.e., the outer surface of the equipment is covered with a light-sensitive polymer resin film, which undergoes a polymerization reaction in UV light, becoming hard and smooth, exerting an anticorrosive effect. For internal and external anticorrosive coatings, the greater the anticorrosive effect, the lower is the score.
Soil corrosiveness	E1	5	Soil corrosiveness is mainly determined on the basis of soil acidity-alkalinity, as well as the testing and monitoring data of microbial flora in soil. If the soil constituent causes soil corrosion and microbial corrosion, 5 points are given.
Corrosiveness of internal medium	E2	5	The corrosiveness of the internal medium is determined by GB/T 30579 [39], engineering practice experience and historical testing data. The higher the corrosiveness, the higher is the score.
Cracking behavior of internal medium	E3	5	The stress corrosion cracking performance of the internal medium is determined by GB/T 30579 [39], engineering practice experience and historical testing data. The greater the possibility of cracking, the higher is the score.
Foundation settlement	E4	5	Foundation settlement is primarily determined by the monitoring data of foundation settlement concerning MST. In case of non-uniform settlement, 5 points are given.
Soil waterlogging/instability	E5	5	Soil waterlogging and instability are mainly determined by the monitoring and inspection data of the MST. In case of soil waterlogging and instability, 5 points are given.
Low-temperature brittle fracture	E6	5	It is determined by the operating conditions of the MST. It does not occur normally but may be caused by hydraulic testing, emergency draining, or permafrost.
Ambient vibration source	E7	5	It is determined by the presence of vibration caused by moving equipment such as a compressor around the MST. If there is a source of vibration, 5 points are given.
Service length	S1	15	It is determined by the service length of the MST. In general, there are 4 service lengths, i.e., 3 years, 10 years, and 15 years. The shorter the service length, the lower is the score.
Historical failure	S2	20	It is determined by daily maintenance and management data, historical inspection, and testing data, etc. In the case of failure, scoring is done according to the failure site and severity. The greater the severity, the higher is the score.
Overall inspection of inner and outer sides	I1	−20	This item ends up with a negative score. It is determined based on whether there was an overall inspection of inner and outer sides in history or there has been an overall inspection of inner and outer sides in the recent inspection period (within 4 years); 20 points are subtracted from the total if both inspections have been conducted, whereas 0 points are subtracted from the total if neither inspection has been conducted.
Risk-based inspection	I2	−10	This item ends up with a negative score. Because of the difficulties involved in the overall inspection on the MST, a risk-based inspection is usually suggested; 10 points are subtracted from the total if it has undergone a risk-based inspection within 4 years of inspection period, otherwise 0 points are subtracted from the total.
Monitoring system	I3	−10	This item ends up with a negative score. It is determined in accordance with the completeness of the MST monitoring system. The more complete the monitoring system, the lower is the score.
Flammability and explosiveness of medium	B1	30	The flammability and explosiveness of the medium are determined in accordance with the content of Appendix A in the group standard T/CPASE GP020-2022 [29]for MST and *The List of Hazardous Chemicals* issued [41] by the State Administration of Work Safety.
Tank volume	V1	30	Tank volume, essentially the mass of the medium in the tank, is used in combination with the flammability and explosiveness of the medium to determine the range of damage caused by explosion to buildings and people. The larger the volume, the higher is the mass of the medium, and the higher is the score. (It can be divided into three classes according to the volumes 2000 m^3^ and 5000 m^3^)
Economic impact	P1	20	Downtime loss and maintenance cost caused to the factory after failure. The higher the cost, the higher is the score.
Environmental effect	L1	20	The impact of medium leakage or explosion on the surrounding environment, groundwater, etc. after failure.

**Fig. 3:**
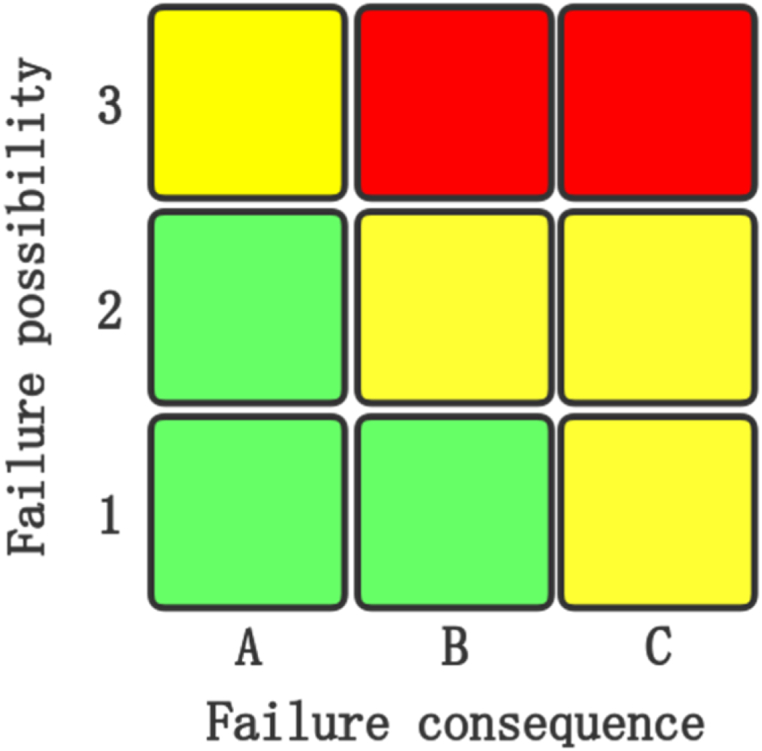
Risk rating matrix for a mounded storage tank.

For the MST, the failure probability and failure consequence are scored according to Table 2, Table 3 The failure probabilities are scored as 1, 2, and 3 if the failure probability scores are <50, 50–80, and >80, respectively. Similarly, with the failure probability scores of <50, 50–80, and >80, the failure consequence is denoted as A, B, and C, respectively. Therefore, as displayed in Fig. 3, a 3 × 3 risk rating matrix can be established to determine the risk level, namely high, medium, or low, of MST. In the figure, green indicates low risk; yellow indicates moderate risk; and red denotes high risk. Support can be provided to enterprises for the integrity management of MST according to the risk level.

We consider a newly commissioned 3300 m^3^ propylene MST as an example for risk calculation. The characteristics of the MST are as follows: material, Q345R; operating temperature, 25 °C; operating pressure, 1.56 MPa; the internal medium, propylene (without H_2_S); MST outer wall was coated with an anticorrosion agent. A continuous sand bed foundation was used, and the surface cladding thickness was 0.8 m. The results of the cladding colony test revealed that the cladding tank was not corrosive. The three sides without process piping in and out were designed as self-combustion slopes for cultivating soil, which allows rainwater to leave naturally, thereby preventing water accumulation. To prevent water accumulation on the top clay, rainwater pipes were pre-buried on the side of the MST. Monitoring systems for settlement, wall thickness, cathodic protection, leakage, were set up to ensure the safe and smooth operation of the MST. Pumps and compressors nearby were used to pressurize the media for the MST.

The failure possibility was calculated as follows:

(1) Material suitability: Based on the GB/T 30579 [39], the medium and material used in the MST were propylene and Q345R, respectively. Propylene is slightly corrosive to the material Q345R in the case of containing trace impurities. According to Table 3, the material suitability can be assigned 5 points; an anticorrosion coating exists on the outside of the MST, but not inside. Therefore, the value assigned to the internal and external anticorrosive coatings is 5 points. In Table 2, the material option score of failure possibility is (material suitability + internal and external anticorrosive coatings) × material weight. For the weights of various sub-items, the managers can set them according to the situation of each device. In this example, the weights were assigned a value of 1. Therefore, the failure probability score is (5+5)×1=10.
(2) In the service environment, the corrosivity of the covering soil can be determined from the monitoring results of the covering soil. No corrosivity can be considered to be 0. The slight corrosivity of internal medium is considered to be 1. Because the internal medium does not contain H_2_S, no cracking is considered to be 0. According to the settlement monitoring data, no foundation settlement was observed in the MST, and the foundation settlement was considered to be 0. The MST was equipped with a perfect drainage system, and the soil waterlogging/instability was considered to be 0. The annual temperature of the service place of the earth-covering tank does not decrease beyond −20 °C; therefore, no low-temperature brittle fracture condition exists, and the low-temperature brittle fracture condition was scored 0. Nearby pumps and compressors are vibration sources, and the ambient vibration source was scored as 5. The weight of service environment was still 1. The service environment score was (0+1+0+0+0+5)×1=6.
(3) Operating state and service length. The MST has been in service for 2 years. According to the classification in Table 3, the MST was the lowest grade of 0–3 years, with a score of 5 points. No failure has been reported since its inception. Therefore, the historical failure was 0. The running state weight was 1, and the running state score was (5+0)×1=5;
(4) Testing and monitoring: Because the MST has been used for 2 years, it is yet to be inspected. Therefore, the MST is yet to be fully inspected internally and externally and based on risk. Therefore, the score of these two items is 0. The MST was equipped with a complete monitoring system and corresponding monitoring devices for water accumulation, corrosion, cathodic protection, and liquid level. Therefore, the score was −10. The testing and monitoring weight was 1. Thus, the test and monitoring score was （0+0−10）×1=−10;

The failure probability was the sum of the above four items, that is, 10+6+5−10=11;

The failure consequences were calculated as follows:

(1) Flammability and explosiveness of medium. The medium of the MST was propylene, which is flammable and explosive. According to Appendix A of the group standard T/CPASE GP020-2022 [29], propylene is in the List of Hazardous Chemicals issued (2022 edition) [41]. Therefore, the value of this item is 30, and the weight is 1. The flammability and explosiveness of the medium score is 30×1=30.
(2) The tank volume, that is, the volume of the MST, was 3300 m^3^, calculated by the filling coefficient of 0.9, and the internal medium was close to 3000 m^3^, which was the middle grade, with a value of 20 points and a weight of 1. Thus, the tank volume score was 20×1=20.
(3) Economic impact: Because the setting of the MST is far away from the main device, and it has relatively perfect measures to mitigate the consequences and corresponding emergency plans, its economic impact is small. Therefore, this item is assigned 5 points, and the weight is 1; thus, the overall economic impact score is 5×1=5.
(4) Environmental effect: Referring to Appendix A of group standard T/CPASE GP020-2022 [13], propylene is slightly toxic, and its leakage may have adverse effects on the surrounding environment and groundwater. Therefore, the score of this item is 10, and the weight is 1. Thus, the environmental effect score is 10×1=10.

The failure consequence is the sum of the above four items, that is, 30+20+5+10=65.

According to the risk-setting conditions, the failure probability of 11 points corresponds to the failure probability of 1, and the failure consequence of 65 points corresponds to the failure consequence of B. The risk matrix in Fig. 3 reveals that the risk level of this earth-covered tank is low.

### 4.3 Monitoring and detection technology for MST

MSTs cannot be detected in the same manner as conventional pressure vessels because conducting a nondestructive inspection on the surface of an MST in a conventional manner is difficult. Furthermore, because an MST is filled with a medium, it cannot be inspected or tested thoroughly on a regular basis. Therefore, an MST undergoes a risk-based inspection based on a configured monitoring system.

Moreover, because an MST is overburdened with a soil layer, it cannot be macroscopically inspected according to the regulations on routine inspection and annual inspection presented in the TSG 21–2016 technical specification [38]. Therefore, at design and installation, measures are typically obtained for state monitoring, such as settlement monitoring, wall thickness monitoring, cathodic protection monitoring, leakage monitoring, and liquid level monitoring. At the operational stage, arrayed grating sensing technology is used for a continuous online measurement of various indexes, such as the operating pressure, temperature, equipment cracks, column base inclination, and overburden settlement. Various online monitoring data are collected to assess the operating state of the equipment and thus satisfy the requirements of routine and annual inspections.

A risk-based inspection of MST is conducted using the aforementioned risk assessment method according to its status, failure mode, failure consequence, management, and maintenance status, that is, an inspection strategy is formulated according to the risk level. First, an internal inspection should be conducted. Novel techniques, such as acoustic emission, crawling robot, or unmanned aerial vehicles, can be used for inspection. Subsequently, the evaluation data should be updated according to the inspection, reworking, and usability evaluation results. This should be followed by risk reassessment. The inspection time is fixed according to the evaluation results and the acceptable level of risk.

### 4.4 Dynamic risk assessment of the MST

The key to the risk assessment of the MST is the accuracy and timeliness of the assessment. For an accurate assessment, the parameters in the assessment must originate from reliable sources and be set up reasonably. Timeliness requires that the risk assessment be dynamically updated in real time, which requires a dynamic risk assessment.

Because the MST body is covered with soil, performing regular inspections and daily testing is not feasible. Therefore, online monitoring and routine maintenance should be conducted to ensure the safe operation of the equipment. With the rapid development of online monitoring technology and its application in the MST, the dynamic risk assessment of the MST can provide rich data support.

Dynamic risk assessment requires enterprises to have a complete set of risk assessment management systems, including the basic information base of the MST, regular inspection information, real-time online monitoring data, and maintenance information. Real-time dynamic data can then be obtained on the MST dynamic risk assessment for subsequent maintenance and management, ensuring timely data support.

## 5 Conclusions and suggestions conclusion

MSTs are widely used in the petrochemical industry for the storage of hazardous chemicals. However, the risk levels associated with their failure are high. Integrity management of MSTs is essential for ensuring their safe operation. Based on the characteristics and risk factors of the MST, first, the integrity management framework system and integrity evaluation method for the MST were established, and the risk assessment method for the MST was innovatively proposed. This method can provide reference and support for the safety management of the MST. However, this study has some shortcomings, such as the modeling work for the fire, explosion, domino effect, and other accidents in the tank area in which the MST is located. These aspects should be improved to enhance the risk assessment of the MST and improve the accuracy of the system. Second, the risk assessment method used to assess the level of risk in this study was qualitative, which can not be as accurate as quantitative risk assessment. With the promulgation and enhancement of laws and standards for MSTs in China, their application in China has increased, and various parameters in the risk assessment can be quantified to conduct quantitative risk assessment.

The study offers following suggestions to improve the integrity management system of MSTs and promote their application:

(1) MST use is in the nascent stage in China. Therefore, an MST information database should be established to collect and analyze MST information, provide data support for the integrity management of MST, or provide support for the construction of a big data-based MST platform in China.
(2) Conducting a routine inspection on MSTs, which are covered with soil and filled with a medium, is difficult. Therefore, strengthening the development of inspection and testing technology for MST is essential, especially the inspection and evaluation technology for non-empty state to ensure the safe operation of MSTs.
(3) Establishing a sound regulation system for MSTs is imperative to promote their rapid application and development in China.

## Funding statement

The authors did not receive support from any organization for the submitted work.

## Data availability statement

No data was used for the research described in the article.

## CRediT authorship contribution statement

**Xiaowei Li:** Writing – original draft, Methodology, Conceptualization. **Chenyang Du:** Writing – review & editing, Writing – original draft. **Chang Liu:** Data curation. **Ce Song:** Supervision. **Jun Yuan:** Writing – review & editing, Supervision. **Jianyu Lu:** Validation. **Yanchao Xin:** Validation.

## Declaration of competing interest

The authors declare that they have no known competing financial interests or personal relationships that could have appeared to influence the work reported in this paper.

## Figures and Tables

**Appendix A:** Common storage media for mounded steel storage vessels

Serial No.	CAS No.	Medium Name	Chemical formula	Toxicity	Combustibility
1	999-81-5	Chlormequat chloride	C_5_H_13_Cl_2_N	Mild harm	stable
2	75-39-8	1-Aminoethylalcohol	CH_3_CH_2_(OH)NH_2_	Mild harm	common
3	102-28-3	*N*-Acetyl-1,3-Phenylenediamine	C_6_H_4_(NH_2_)NHCOCH_3_	Mild harm	common
4	122-80-5	*P*-Aminoacetanilide	C_6_H_4_(NH_2_)NHCOCH_3_	Mild harm	common
5	108-46-3	Resorcinol	C_6_H_4_(OH)_2_	Mild harm	common
6	100-52-7	Benzaldehyde	C_6_H_5_CHO	Mild harm	common
7	107-03-9	1-Propyl mercapton	CH_3_CH_2_CH_2_SH	Mild harm	easy
8	67-64-1	Acetone	CH_3_COCH_3_	Mild harm	easy
9	78-82-0	Isobutyronitrile	C_8_H_12_N_4_	Mild harm	common
10	121-57-3	*p*-Aminobenze nesulfonic acid	C_6_H_4_(NH_2_)(SO_2_OH)	Mild harm	common
11	10605-21-7	Carbendazim	C_6_H_4_(N)(NH)–C–NHCOOCH_3_	Mild harm	common
12	75-37-6	1,1-Difluoroethane	CH_3_CHF_2_	Mild harm	easy
13	127-19-5	Dimethylacetamide	CH_3_CON(CH_3_)_2_	Mild harm	common
14	583-78-8	2,5-Dichlorophenol	C_6_H_3_Cl_2_OH	Mild harm	common
15	119-26-6	2,4-Dinitrophenylhydrazine	(NO_2_)_2_C_6_H_2_NHNH_2_	Mild harm	easy
16	109-89-7	Diethylamine	(CH_3_CH_2_)_2_NH	Mild harm	easy
17	579-66-8	2,6-Diethylaniline	NH_2_C_6_H_3_(CH_2_CH_3_)_2_	Mild harm	common
18	462-06-6	Fluorobenzene	C_6_H_5_F	Mild harm	easy
19	78-10-4	Tetraethyl orthosilicate	(CH_3_CH_2_O)_4_Si	Mild harm	easy
20	67-56-1	Methanol	CH_3_OH	Mild harm	easy
21	100-61-8	N-Methylaniline	C_6_H_5_NHCH_3_	Mild harm	easy
22	108-39-4	m-Cresol	CH_3_C_6_H_4_OH	Mild harm	common
23	60-51-5	Rogor	(CH_3_O)_2_P(S)SCH_2_C(O)NHCH_3_	Mild harm	easy
24	126-73-8	*Tri*-*n*-butylphosphate	(CH_3_CH_2_CH_2_CH_2_O)_3_PO	Mild harm	common
25	88-75-5	*o*-Nitrophenol	NO_2_C_6_H_4_OH	Mild harm	easy
26	67-72-1	Hexachloro ethan	CCl_3_CCl_3_	Mild harm	cannot
27	100-97-0	Hexamethylenetetramine	(CH_2_)_6_N_4_	Mild harm	common
28	108-90-7	Chlorobenzene	C_6_H_5_Cl	Mild harm	easy
29	108-42-9	*m*-Chloroaniline	ClC_6_H_4_NH_2_	Mild harm	common
30	87-60-5	3-Chloro-2-methylaniline	CH_3_C_6_H_3_(NH_2_)Cl	Mild harm	common
31	90-13-1	α-Chloronaphtalene	C_10_H_7_Cl	Mild harm	common
32	121-87-9	2-Chloro-4-nitroaniline	C_6_H_3_(NH_2_)(NO_2_)Cl	Mild harm	common
33	89-63-4	4-Chloro-2-nitroaniline	C_6_H_3_(NH_2_)(NO_2_)Cl	Mild harm	common
34	134-32-7	1-Naphthylamine	C_6_H_4_C_4_H_3_NH_2_	Mild harm	common
35	90-15-3	1-Naphthol	C_6_H_4_C_4_H_3_OH	Mild harm	common
36	51630-58-1	Phenvalerate	C_6_H_5_–*O*–C_6_H_4_–CH(CN)(O–COCH-(CH(CH_3_)_2_)(C_6_H_4_Cl)	Mild harm	easy
37	41814-78-2	tricyclazole	C_9_H_7_N_3_S	Mild harm	common
38	75-50-3	Trimethylamine	(CH_3_)_3_N	Mild harm	easy
39	108-78-1	Melamine	(CN)_3_(NH_2_)_3_	Mild harm	cannot
40	120-82-1	1,2,4-Trichlorobenzene	C_6_H_3_Cl_3_	Mild harm	common
41	21757-82-4	Plifenate	Cl_2_C_6_H_3_–CH(OCOCH_3_)(CCl_3_)	Mild harm	common
42	102-71-6	Triethanoamine	(C_2_H_5_)_3_SnCl	Mild harm	common
43	1129-41-5	Tsumacide	CH_3_C_6_H_4_OCO–NH–CH_3_	Mild harm	common
44	1314-80-3	Phosphorus pentosulfide	P_2_S_5_	Mild harm	easy
45	82-68-8	Pentachlorobenzene	C_6_Cl_5_NO_2_	Mild harm	common
46	63-25-2	Carbaryl	C_6_H_4_C_4_H_3_OCONHCH_3_	Mild harm	common
47	88-74-4	o-Nitroaniline	C_6_H_4_(NH_2_)NO_2_	Mild harm	common
48	99-09-2	m-Nitroaniline	C_6_H_4_(NH_2_)NO_2_	Mild harm	common
49	88-72-2	o-Nitrotoluene	CH_3_C_6_H_4_NO_2_	Mild harm	easy
50	89-62-3	4-Methyl-2-nitroaniline	CH_3_C_6_H_3_(NH_2_)NO_2_	Mild harm	common
51	100-00-5	*p*-Chloronitrobenzene	ClC_6_H_4_NO_2_	Mild harm	common
52	74-96-4	Bromoethane	CH_3_CH_2_Br	Mild harm	common
53	98-83-8	Isopropylbenzene	C_6_H_5_CH(CH_3_)_2_	Mild harm	easy
54	26087-47-8	Iprobenfos	C_13_H_21_O_3_PS	Mild harm	common
55	78-93-3	2-Butanone	CH_3_COH_2_CH_3_	Mild harm	easy
56	60-29-7	Ether	CH_3_CH_2_OCH_2_CH_3_	Mild harm	easy
57	30560-19-1	Acephate	(CH_3_O)(CH_3_S)(CH_3_OCNH)P–O	Mild harm	common
58	3766-81-2	Fenobucarb	C_12_H_17_NO_2_	Mild harm	easy
59	115-07-1	Propylene	CH_2_CHCH_3_	Mild harm	easy
60	74-98-6	Propane	CH_3_CH_2_CH_3_	Mild harm	easy
61	106-97-8	Butane	CH_3_CH_2_CH_2_CH_3_	Mild harm	easy
62	106-98-9	1-Butane	CH_3_CH_2_CHCH_2_	Mild harm	easy
63	74-85-1	Ethylene, Ethene	CH_2_CH_2_	Mild harm	easy
64	75-28-5	Isobutane	CH_3_CH(CH_3_)CH_3_	Mild harm	easy
65	8030-30-6	Naphtha	C_4_C_6_烷烃	Mild harm	easy
66	68476-85-7	Liquefied petroleum gas	C_3_C_4_混合烃	Mild harm	easy
